# The Cause of the Pyrexia in Fever

**Published:** 1901-02-02

**Authors:** 


### THE CAUSE OF THE PYREXIA IN FEVER.

To dream and speculate on topics connected with pathology may not always be profitable—indeed, if given too free a rein it is a pastime which easily leads to error. Yet if one could do it with any approach to certainty what interest it would add to one's medical work to be able to picture in one's mind just what is going on within the patient's frame! Dr. T. J. Maclagan[1] has drawn a picture of the events of typhoid fever which is certainly provocative of thought, and is worthy of most careful consideration, even though it may not be true. If it were true it would not only explain many of the phenomena of typhoid fever, but would have to be applied to fevers of many other kinds. We may, however, say at once that we doubt about the fever process being quite so simple an affair as Dr. Maclagan suggests. What he says in regard to heat-production has a strong air of probability, but it does not touch the other equally important factor in the production of pyrexia, derangement in the mechanism of heat-loss. He points out that the most prominent of the general symptoms of typhoid fever, that which from first to last is the dominant feature of the illness, is fever—that is, a rise in the temperature of the body. This, he says, in uncomplicated cases, reaches its maximum before the tenth day, and before the local lesion has given evidence of its existence. How, then, he asks, does the bacillus produce this general febrile disturbance? A local lesion which does not cause enough local disturbance to enable us to diagnose its existence is not likely to cause so much general febrile disturbance as is noted at the end of the first week of typhoid fever. It cannot indeed be held that so important a lesion causes no general disturbance, and has no share in the production of the fever, but it is evident that its presence is not sufficient to explain the marked rise of temperature which occurs during the first week of the disease; and to explain this he turns to the vital processes involved in the growth of the enormous multitude of germs which he holds are at this time being poured into the circulation to live their lives and to increase and multiply in all parts of the patient's body. These, he says, are not mature bacilli, but young germs which have just been reproduced, have just entered on their separate existence, and whose organic growth has yet to be completed. "It is the action resulting from the organic growth of these millions of organisms that gives rise to the general febrile disturbance associated with the development of the bowel lesion."

When we examine minutely the various phenomena of the febrile state as it occurs in the specific fevers, we find, he says, that they ultimately resolve themselves into two—increased consumption of nitrogen and increased consumption of water—and from these two all the others result. We come, then, to this, that the cause of the wasting of the nitrogenous tissues is the consumption by millions of bacilli of the nitrogen destined for their nutrition, and that the cause of the thirst, dry skin, and scanty urine is the consumption by the same bacilli of the water destined for the tissues, while the rise of temperature and the quickening of the circulation result from the increased metabolism arising from this action going on in the minute structure of the nitrogenous tissues.

But to make the picture complete we must frame some hypothesis to account for the fever ever coming to an end. We are told then that the fever comes to an end when the bacilli cease to be reproduced, and they cease to be reproduced when the nidus is exhausted; that is, when all the glands which form the nidus have been destroyed. The course of events shows that on an average it takes five weeks, more or less, for the completion of this process—two weeks occupied by the period of incubation and three by the febrile stage. How, then, about relapses? As to this, one has to suppose that not all the intestinal glands are affected in a first attack of typhoid fever: some remain untouched, and these may become affected later. They in turn may again pour into the system their shoals of fry, again to develop in the midst of the tissues, and again to go on causing pyrexia until they in turn cease to be poured out in consequence of their nidus also being destroyed; all of which is very well, and as a pretty mental picture it is very well indeed. But whether in the first week of typhoid the whole system is as a fact infested with these bacilli; whether these bacilli growing in the intestinal glands do as a fact give off shoals of germs instead of multiplying by division; whether the mere increase of heat production by the growth of these bacilli would of itself account for the pyrexia; and, finally, whether a theory is to be regarded as acceptable which requires us to believe that these bacilli go on being reproduced in the tissues, so long as it is a question of a rise of temperature, but when it becomes a question of the cessation of the fever, attributes this to the cessation of the supply of germs from the nidus in the intestinal glands; these are questions into which we will not at this moment enter. But, as put by Dr. Maclagan, it all looks very simple, which is the provoking part of many a day-dream.

[1] Lancet, Jan. 12.

